# Purification and Characterization of a Biofilm-Degradable Dextranase from a Marine Bacterium

**DOI:** 10.3390/md16020051

**Published:** 2018-02-07

**Authors:** Wei Ren, Ruanhong Cai, Wanli Yan, Mingsheng Lyu, Yaowei Fang, Shujun Wang

**Affiliations:** 1 Jiangsu Marine Resources Development Research Institute, Huaihai Institute of Technology, Lianyungang 222005, China; renwei1004570447@163.com(W.R.); yanwanli2016@163.com (W.Y.); mslu@hhit.edu.cn (M.L.); foroei@163.com (Y.F.); 2 Key Laboratory of Marine Biology, Nanjing Agricultural University, Nanjing 210000, China; crh1987@163.com; 3 Co-Innovation Center of Jiangsu Marine Bio-industry Technology, Huaihai Institute of Technology, Lianyungang 222005, China; 4 State Key Laboratory of Marine Environmental Science, College of Ocean and Earth Sciences, Xiamen University, Xiamen 361005, China; 5 College of Marine Life and Fisheries, Huahai Institute of Technology, Lianyungang 222005, China

**Keywords:** marine agent, *Catenovulum*, alkaline and cold-adapted dextranase, isomaltoogligosaccharides, biofilm, dental caries

## Abstract

This study evaluated the ability of a dextranase from a marine bacterium *Catenovulum* sp. (Cadex) to impede formation of *Streptococcus mutans* biofilms, a primary pathogen of dental caries, one of the most common human infectious diseases. Cadex was purified 29.6-fold and had a specific activity of 2309 U/mg protein and molecular weight of 75 kDa. Cadex showed maximum activity at pH 8.0 and 40 °C and was stable at temperatures under 30 °C and at pH ranging from 5.0 to 11.0. A metal ion and chemical dependency study showed that Mn^2+^ and Sr^2+^ exerted positive effects on Cadex, whereas Cu^2+^, Fe^3+^, Zn^2+^, Cd^2+^, Ni^2+^, and Co^2+^ functioned as inhibitors. Several teeth rinsing product reagents, including carboxybenzene, ethanol, sodium fluoride, and xylitol were found to have no effects on Cadex activity. A substrate specificity study showed that Cadex specifically cleaved the α-1,6 glycosidic bond. Thin layer chromatogram and high-performance liquid chromatography indicated that the main hydrolysis products were isomaltoogligosaccharides. Crystal violet staining and scanning electron microscopy showed that Cadex impeded the formation of *S. mutans* biofilm to some extent. In conclusion, Cadex from a marine bacterium was shown to be an alkaline and cold-adapted endo-type dextranase suitable for development of a novel marine agent for the treatment of dental caries.

## 1. Introduction

Dextranases (α-1,6-d-glucan-6-glucanohydrolase; EC 3.2.1.11) hydrolyze dextran to oligosaccharides at the α-1,6 glucosidic bond, and are widely used in medical, dental, and sugar industries. In clinical applications, specific clinical dextran produced by dextranase can be used as a blood substitute in emergencies [1,2,3]. In the sugar industry, dextranase has been used to resolve the poor clarification and throughput that dextran can cause in sugarcane juice [4,5,6,7]. It is worth noting that this enzyme can be used with commercial dextran to directly synthetize isomaltose and isomaltooligosaccharides which exhibit prebiotic effects [8,9,10]. A previous report proposed that dextranase may be capable of treating dental plaques [2,3,11]. For this reason, the use of dextranase to treat dental caries has attracted a great deal of attention, particularly with respect to the degradation of dextran in dental plaques. Bacterial cells form biofilms as a protective barrier from external conditions, serving as a mechanism for improving survival and dispersion [12,13]. *Streptococcus mutans* is the main cause of dental decay in human teeth and key modulator of the development of cariogenic biofilms [14,15]. Accumulation of this cariogenic bacterium within the biofilm may lead to the onset of periodontal inflammation. Thus, dislodging the biofilm is the main therapy for periodontal inflammation.

An alkaline dextranase may be suitable for the treatment of dental caries because alkaline tooth-rinse products are expected to be more amenable to enamel than acidic products [16]. In addition, dextranase works efficiently at temperatures of about 37 °C and may also contribute to the degradation of human dental plaques. However, the most common source of dextranase, fungi, produce many acidic and megathermal dextranases, which catalyze at pH values ranging from pH 5.0 to 6.5 and temperatures above 50 °C, and are unstable under alkaline conditions. Dextranases are seldom capable of catalysis under both alkaline and moderate-temperature conditions [1]; however, enzymes from marine microorganisms may be an exception [17,18,19].

In this study, a dextranase produced by a marine bacterium *Catenovulum* sp. DP03 (CGMCC No. 7386) was sequenced and selected for extracellular experiments [20]. This study demonstrated a method for purifying, characterizing and hydrolyzing products of dextranase from the marine strain DP03 and analyzed its effects on *S. mutans* biofilm. The results provide insights into additional applications for this enzyme.

## 2. Results

### 2.1. Purification of Dextranase

**Figure 1:**
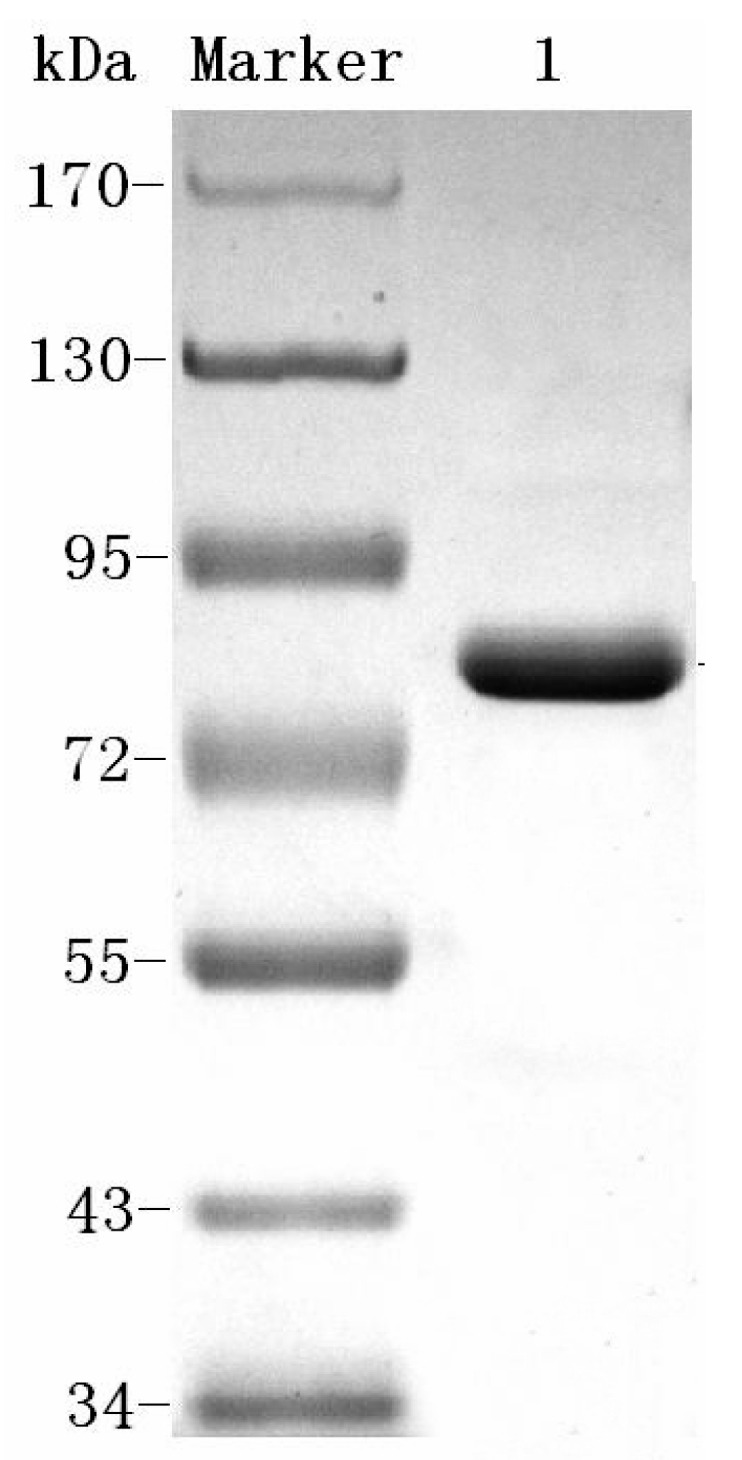
Sodium dodecyl sulfate-polyacrylamide gel electrophoresis (SDS-PAGE) of Cadex. Lane Maker: protein marker. Lane 1: purified Cadex.

A summary of the efficient extraction protocol used to purify *Catenovulum* sp. DP03 dextranase (hereafter called Cadex), which included ultrafiltration, ethanol precipitation, ammonium sulfate precipitation, and ion exchange chromatography (Table 1). The activity of crude dextranase was 77.9 U/mg, and the activity of Cadex sequentially purified by ultrafiltration, ethanol precipitation, and ammonium sulfate precipitation was 163.5 U/mg, 223.3 U/mg and 341.6 U/mg, respectively. When ion exchange chromatography was used for purification, Cadex was purified 29.6-fold with a specific activity of 2309 U/mg protein and a yield of 16.9%. About 5.4% of enzymatic activity was lost and specific enzyme activity was increased by 48.7% after ultrafiltration. Ethanol precipitation and ammonium sulfate precipitation were used to remove polysaccharides and proteins. Ion exchange chromatography was performed to optimize the purification. Ten fractions containing proteins were eluted from Q-sepharose column, among which only one fraction showed dextranase activity. A Q-sepharose column with gradient elution of 45% 800 mM NaCl is an efficient method for Cadex purification. Finally, a homogenetic dextranase was purified. As shown in Figure 1, Cadex appeared as a single band of an estimated molecular weight of 75 kDa. The isoelectric point (pI) was 5.0.

### 2.2. Characterization of Dextranase

#### 2.2.1. Effects of pH and Temperature on Dextranase Activity

**Figure 2:**
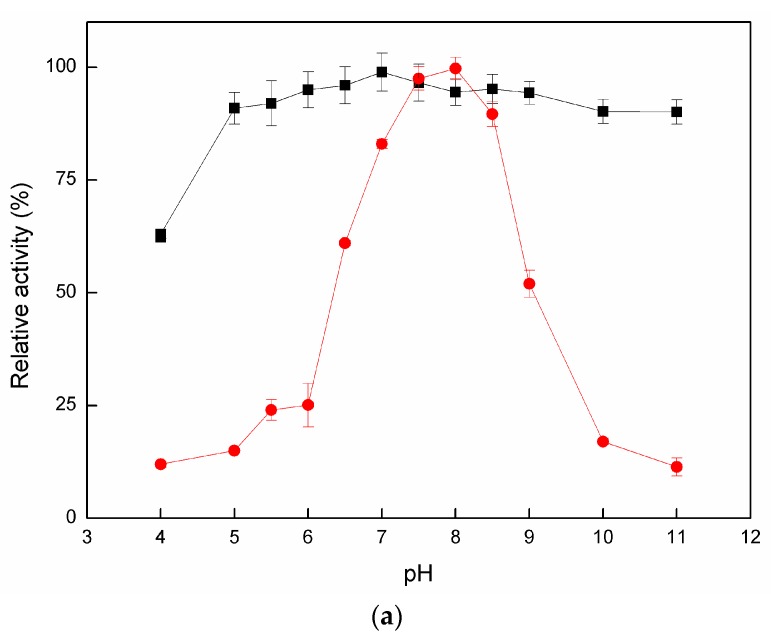

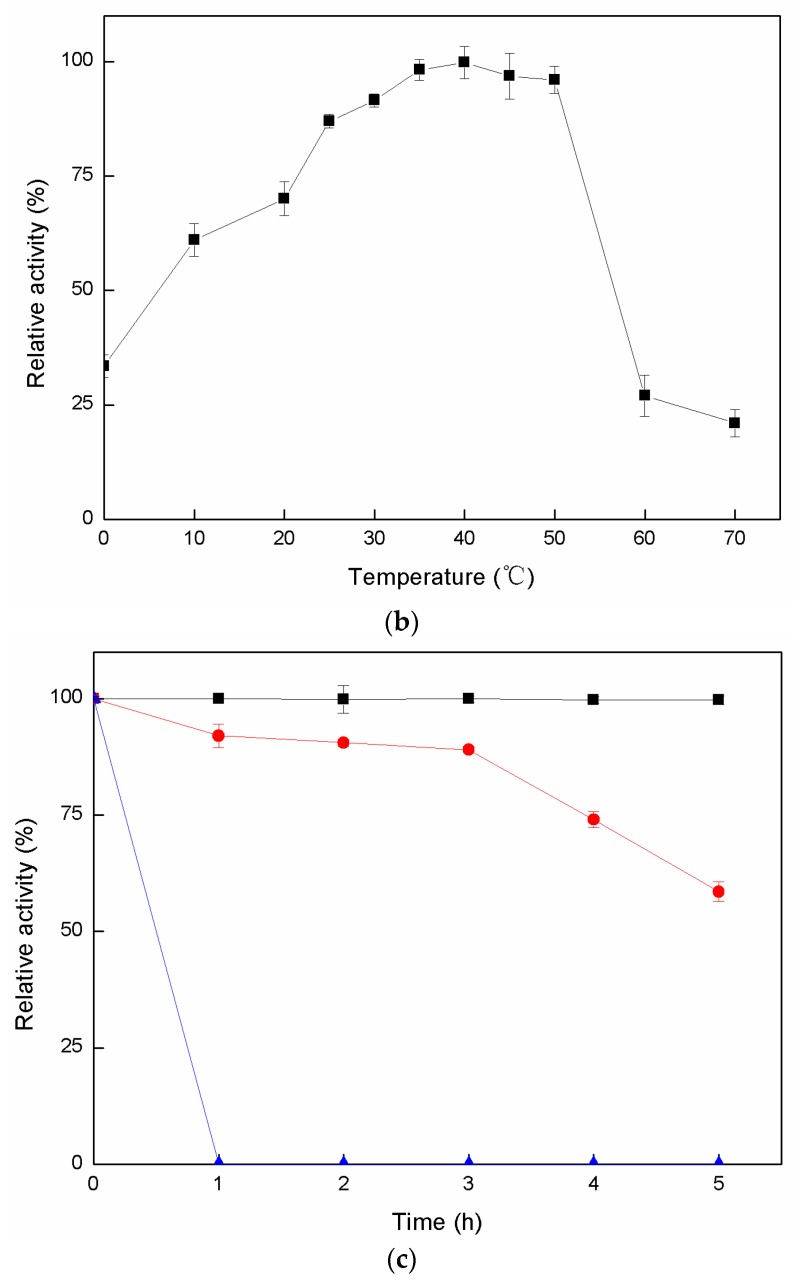
(**a**) Optimum pH and stability curves of Cadex. For each pH, activity was assayed at 40 °C and considered relative activity (●). The pH stability curve (∎) represents residual activity after pre-incubation for 1 h at 25 °C. (**b**) Effects of temperature on the activity. (**c**) Effects of temperature on thermal stability of Cadex. Thermal stability: ∎ 30 °C, ● 40 °C, ▲ 50 °C.

The optimum pH for maximum Cadex activity was 8.0 within the range of pH 7.0–9.0 (Figure 2a). The effects of temperature on dextranase activity are shown in Figure 2b. The optimal temperature for dextranase activity was 40 °C within the range of 25–50 °C, showing peak relative activity no less than 87%. However, dextranase activity decreased up to 60 °C. Cadex also showed 33.8% relative activity at 0 °C.

#### 2.2.2. Enzymatic Stability

Figure 2a shows that Cadex retained more than 90% activity within a pH range of 5–11 (100% activity at pH 7.0). Purified Cadex showed thermal stability at 30 °C (pH 7.5) for 5 h. Under these conditions, almost 100% activity was retained. At 40 °C, 40% of activity was lost after 5 h of exposure. Above 50 °C, activity rapidly declined to undetectable levels at 1 h (Figure 2c).

#### 2.2.3. Effects of Metal Ions and Reagents on Dextranase Activity

Alkaline proteases from microorganism require a divalent cation such as Ca^2+^, Mg^2+^, and Mn^2+^ or a combination of these cations, for maximum activity [19]. The effects of various metal ions on Cadex were investigated and the results are shown in Table 2. Enzyme activity was slightly increased upon exposure to 1 mM and 5 mM MgCl_2_ or SrCl_2_, respectively. This is consistent with previous observations [21,22]. However, enzyme activity was inhibited by exposure to 1 mM CuCl_2_, FeCl_3_, ZnCl_2_, CdCl_2_, NiCl_2_, or CoCl_2_, and even more so at concentrations of 5 mM, consistent with the results from another report [23], although these data conflict with the results reported by Birol et al. [24]. No apparent effects on dextranase activity were observed after exposure to BaCl_2_, NH_4_Cl, CaCl_2_, KCl, or LiCl. However, the cations Co^2+^ and Ca^2+^ had the opposite effects on dextranase from *Chaetomium erraticum* and *Arthrobacter oxydans* KQ11 [3,10]. The effects of several types of chemical treatments for dental caries on the activity of Cadex are shown in Table 3. The data indicate that 5% ethanol, 0.1% carboxybenzene, sodium fluoride and xylitol had no apparent effects on the Cadex activity.

#### 2.2.4. Substrate Specificity and the Hydrolysis Products of Dextranase

**Figure 3:**
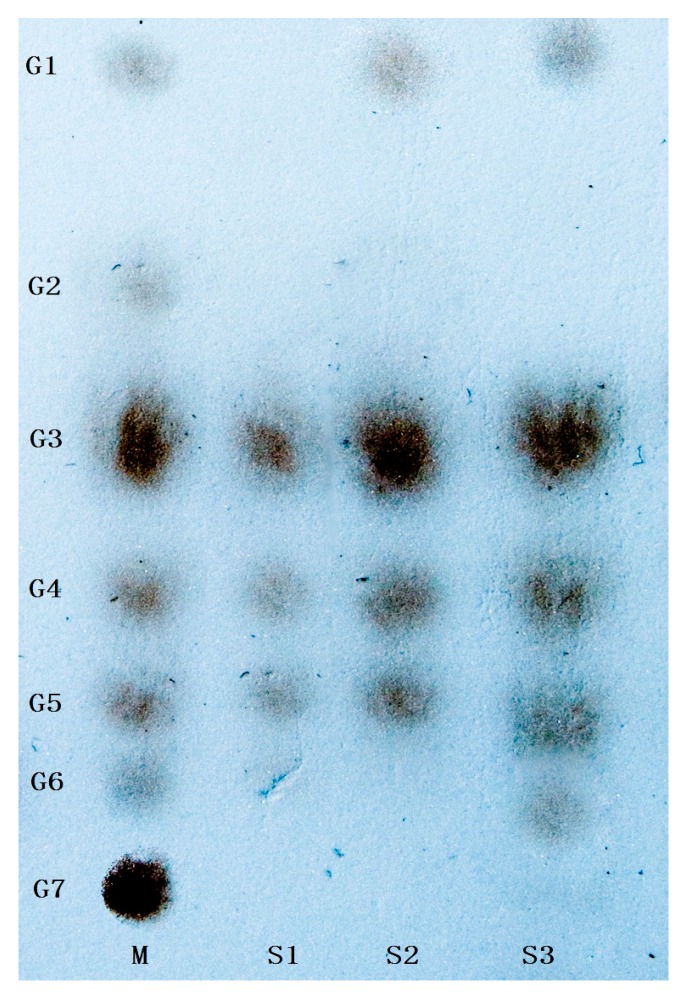
Thin-layer chromatogram of the products from Cadex. Symbols: G1 to G7 a series of authentic sugar standards of glucose, maltose, isomalto-triose, isomalto-tetraose, isomalto-pentaose, isomalto-hexaose, and isomalto-heptaose, respectively. M is the standard marker, S1 to S3 show the 3 h, 24 h, and 72 h reaction times, respectively.

Dextranase activity in the catalyzed hydrolysis of carbohydrates with different glucosidic linkages was determined and used as an indicator of the substrate specificity of Cadex (Table 4). Dextranase showed high specificity towards dextrans containing the α-1,6 glucosidic bond. The poor hydrolysis of soluble starch might indicate that Cadex can only cleave α-1,6 glycosidic bond. Soluble starch was formed using only a few α-1,6 glucosidic and α-1,4 glucosidic linkages [25]. No activity was detected in pullulan, which was mainly formed by α-1,4 glucosidic linkages. The same results were observed with chitin and cellulose, which are formed by β-1,4 glucosidic linkages. First, isomalto-triose (G3), isomalto-tetraose (G4), isomalto–pentaose (G5) and higher molecular weight maltooligosaccharides were found to be the products after a 3 h reaction. The hydrolysis products after a 24 h reaction were glucose (G1), G3, G4, and G5, and G6 was an extra sugar product when the reaction time was extended to 72 h, as shown by thin-layer chromatography (TLC) (Figure 3).

**Figure 4:**
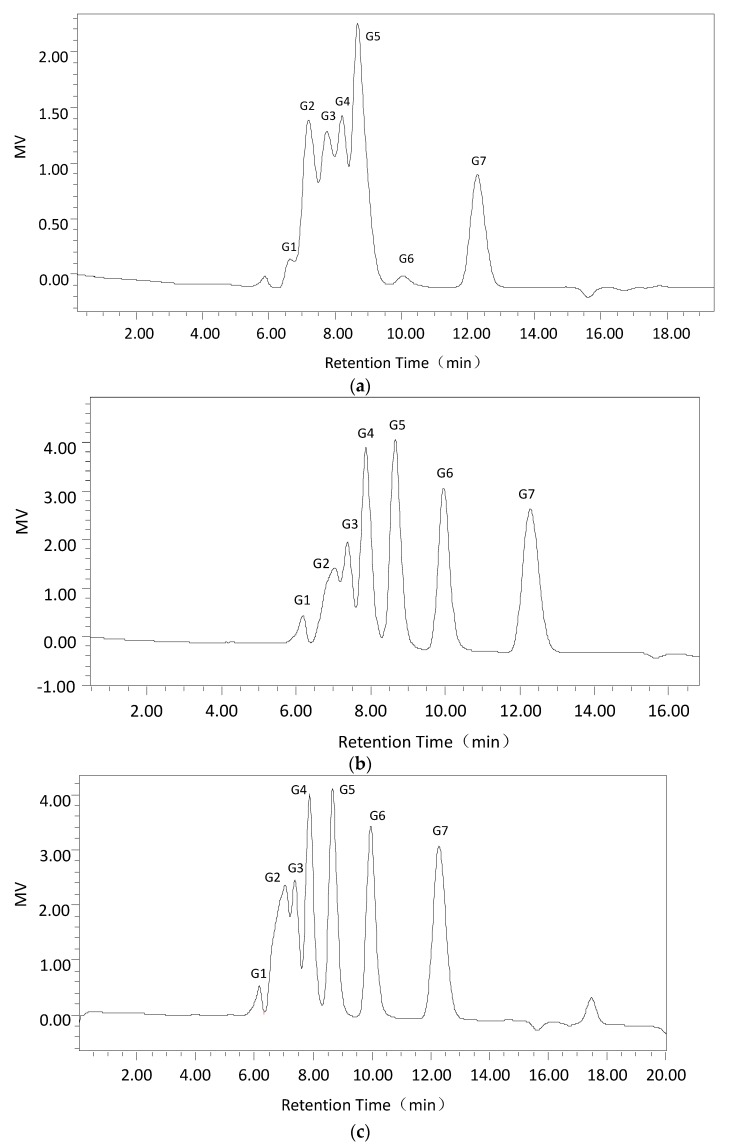

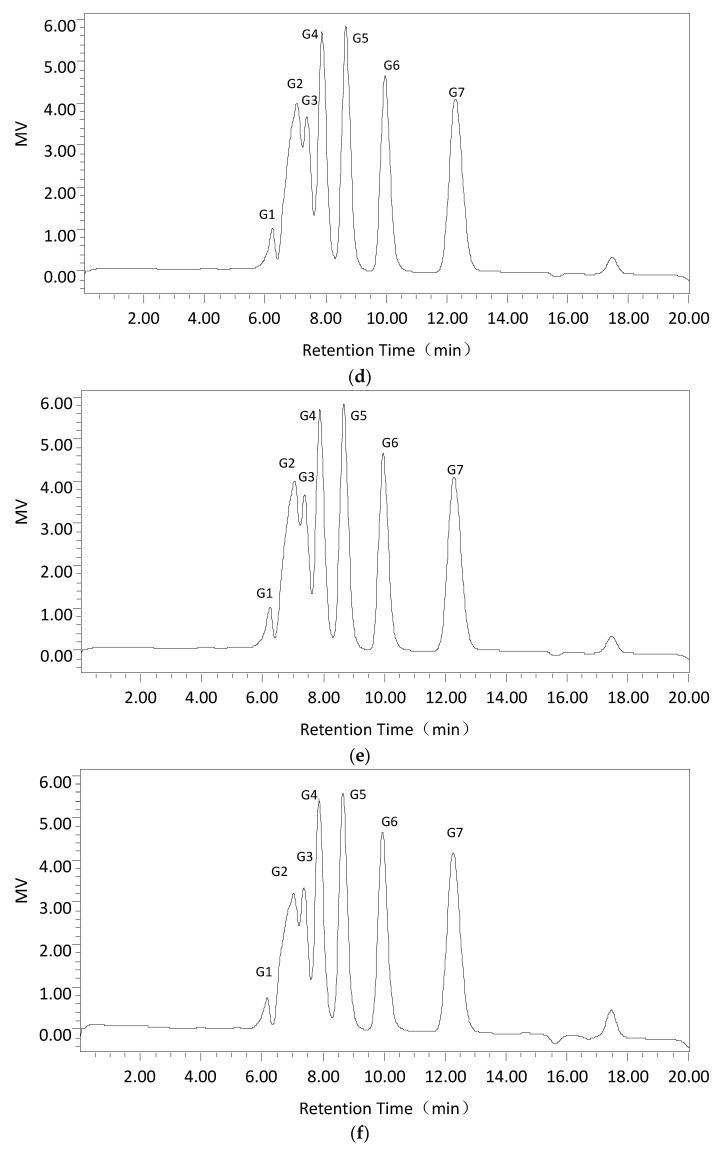
The 3% dextran T70 was treated at 40 °C and pH 8.0 for different periods with the products measured by HPLC: (**a**) the results for standards (G1 to G7 a series of authentic sugar standards of glucose, maltose, isomalto-triose, isomalto-tetraose, isomalto-pentaose, isomalto-hexaose, and isomalto-heptaose, respectively); and (**b**–**f**) the results for 3% dextran T70 treated for 15 min, 30 min, 1 h, 3 h, and 5 h with Cadex.

Second, high-performance liquid chromatography (HPLC) indicated that isomaltooligomers were the main products released by Cadex regardless of reaction time (Figure 4). Each hydrolyzed product was quantified by Empower GPC software (Table 5). G1, G2, G3, G4, G5, G6, and G7 were products of glucose, maltose, isomalto-triose, isomalto-tetraose, isomalto-pentaose, isomalto-hexaose, and isomalto-heptaose, respectively. The results indicated that a small amount of glucose was detected, which is similar to most fungal dextranases. They maintained a dynamic equilibrium, which did not increase with longer reaction times. Moreover, the amounts of maltose, isomalto-triose and isomalto-tetraose slightly declined, and an increase in time from 15 min to 5 h did not increase the yield. However, isomalto-hexaose and isomalto-heptaose accumulated more quickly than the other sugars as the time increased from 15 min to 5 h. At the same time, the yield of these intermediate high molecular weight oligomers (15–20%) was significantly higher than that of glucose (about 2%). This was similar to other reports in which macromolecule isomaltooligomers accumulated in the first period (6 h), and the other sugars were hydrolyzed to produce other smaller sugars [26]. The isolation of small amounts of glucose and some intermediate high molecular weight oligomers seemed to be random rather than through the stepwise hydrolysis of polysaccharide, making Cadex an endo-type dextranase.

Sugars were identified and measured by HPLC. The hydrolysis condition yielded 3% dextran T70 at 40 °C and pH 8.0.

### 2.3. Effects of Cadex on Biofilm

**Figure 5:**
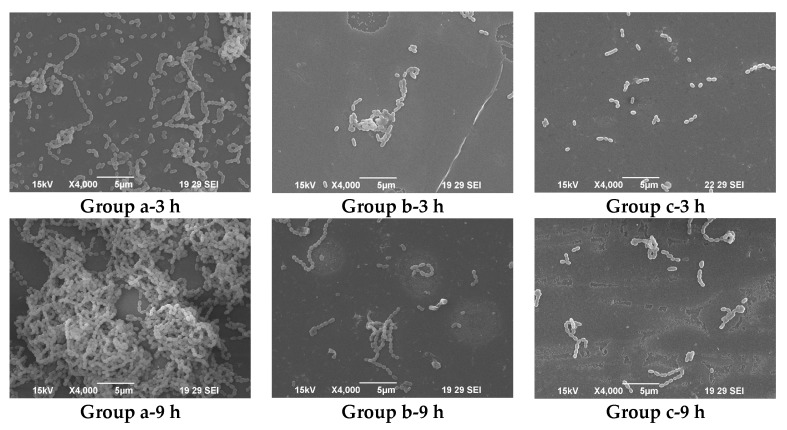

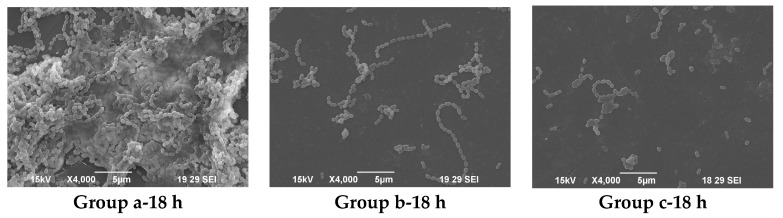
Electron microscopy of *S. mutans* biofilm formed on glass coverslips in the presence and absence of dextranase at different periods: (**Group a**) blank control, note the equal volume of cell-free pure water was added to replace dextranase; (**Group b**) biofilm subjected to 40 U/mL *Penicillium dextranase*; and (**Group c**) biofilm subjected to 35 U/mL Cadex.

Table 6 shows the comparison of biofilm inhibitory rates between *Penicillium* dextranase and Cadex under various concentrations. It can be clearly seen that both *Penicillium* dextranase and Cadex impeded the biofilm formation. The minimum biofilm inhibitory concentration for 90% inhibition (MBIC_90_) was calculated when 40 U/mL *Penicillium* dextranase and 30 U/mL Cadex were added to the media after which the effects of the dextranases on *S. mutans* biofilm formation were analyzed. Cadex was more efficient than *Penicillium* dextranase in inhibiting *S. mutans* biofilm formation. Figure 5 shows the bacterial morphology and biofilm, as observed by scanning electron microscopy (SEM.). In the blank control group, *S. mutans* grew well and the biofilm developed smoothly with prolonged time. At 18 h, a thick biofilm with dense cells were seen, with no obvious structural breakdown. In contrast, biofilm did not form easily when *S. mutans* was cultured in brain heart infusion (BHI) medium by adding the MBIC_90_ of Cadex or *Penicillium* dextranase. Dextranase impeded biofilm formation and reduced the number of *S. mutans* cells that adhered to the glass coverslips. Cadex had inhibitory effects on *S. mutans* biofilm formation. A previous report proposed that crude *Catenovulum* dextranase can prevent *S. mutans* from forming biofilms, however, it used crude dextranase and was a preliminary assessment [20].

## 3. Discussion

A psychrotolerant dextranase-producing bacterium named *Catenovulum* sp. DP03 was previously studied [20]. However, to the best of our knowledge, this is the first report of the purification and characterization of dextranase from *Catenovulum.* Purification of crude dextranase by ammonium sulfate fractionation and Sepharose 6B chromatography, which resulted in a 6.69-fold increase in specific activity and an 11.27% recovery, was previously reported [23]. This system of the aforementioned procedure may be used to produce homogenetic dextranase. The process can easily be scaled up and is cost-effective. The molecular weight of Cadex was about 75 kDa, which resembled that of dextranase from *Sporotrix schencki* (79 kDa) [27]. Bacteria producing dextranases generally have molecular weights ranging from 60 to 114 kDa [28,29,30]. The smallest dextranase (23 kDa) is from *Lipomyces starkeyi* [24], and the largest (175 kDa) is from *Streptococcus sobrinus* [31].

Endo-type Cadex showed high specificity towards dextrans containing α-1,6 glucosidic bonds. Moreover, the main hydrolysis products of Cadex were isomaltooligomers [30,32,33]. Dextranase from *Chaetomium* [34], *Aspergillus* [35], *Penicillium* [36], and *Fusarium* [37] synthesize comparatively low amounts of glucose and higher amounts of isomaltooligosaccharides. Isomaltooligosaccharides can promote the growth and proliferation of *Bifidobacteria* and *Lactobacillus* [1,38]. Numerous isomaltooligosaccharides are prebiotics, which are produced endodextranases and have garnered much commercial interest [33].

The optimum pH for Cadex activity tends to be alkaline, and recently reported alkalophilic cases were *Streptomyces* sp. NK458 and *Bacillus subtilis* NRC-B233b, which had maximum activities at pH 9.0 and pH 9.2 [7,39]. Evidence is accumulating that alkali generation plays a major role in pH homeostasis which may modulate the initiation and progression of dental caries [40]. Therefore, alkalophilic Cadex may be suitable for the development of novel marine agents for the treatment of this condition [16]. Cadex had catalytic efficiency at 0 °C, similar characteristics to other cold-adapted enzymes: for example, a cold-adapted ι-carrageenase showed 36.5% relative activity at 10 °C [41] and a cold-adapted β-glucosidase retained more than 60% of its activity at temperatures ranging from 15 °C to 35 °C [42]. Cold-adapted enzymes have optimal catalyst temperatures near 30 °C and remain efficient at 0 °C. Cadex can be classified as a cold-adapted enzyme according to the system developed by Margesin and Schinner [43]. The excellent pH stability of Cadex distinguishes it from other dextranases, which are generally unstable across a broad pH range [1,7,22,27]. It would be easier to hydrolyze dextran than dextranases in acid/alkaline catalysis conditions. We speculate that Cadex may be suitable for widespread use. We have classified Cadex as a cold-adapted dextranase, which may explain its lower thermo-stability than terrestrial dextranases [1]. Nevertheless, in our early studies, crude Cadex showed greater thermostability than purified dextranase, as it was stable at 45 °C, and its half-life was 10 h (data not shown).

The present study proposes that purified *Catenovulum* dextranase, namely Cadex, is an alkalophilic and cold-adapted dextranase that is considered to be a novel marine dextranase of dealing with biofilms. The failure of biofilm formation is attributable to the failure of extracellular polysaccharides to form efficiently, possibly due to cleavage of the α-1,6 glucosidic linkages in the biofilm that occurs in the presence of Cadex. The oral *Streptococcus* biofilm is formed by α-(1,3)-glucan and α-(1,6)-glucan, in which the α-1,6 glucosidic linkages are degradable by dextranase while the α-1,3 glucosidic linkages can be cleaved by mutanase [44]. *Penicillium* dextranase is often used as the standard dextranase in studies of this enzyme such as studies showing dextran removal during sugar manufacturing [7]. Cadex was a favorable biofilm inhibitor that surpassed the inhibitory ability of *Penicillium* dextranase at the same concentration. In addition, common teeth rinsing products, such as carboxybenzene, ethanol, sodium fluoride, and xylitol, had no negative effects on Cadex activity. Marine organisms are regarded as a prolific resource of novel bioactive metabolites, including a vast array of macrolides, cyclic peptides, pigments, polyketides, terpenes, steroids and alkaloids [45]. At the same time, marine enzymes are important bioactive metabolites which characterized by high salt tolerance, hyperthermostability, and low ideal temperature tolerance. These beneficial properties make Cadex an attractive candidate for development as a novel reagent for dental plaque treatment [46,47].

## 4. Materials and Methods

### 4.1. Chemicals

Q-Sepharose FF, dextran (T20, T40, T70, and T500), and PhastGel IEF 3-9 were obtained from GE Healthcare (Uppsala, Sweden). A prestained protein PAGE ruler was obtained from Fermentas (Waltham, MA, USA). An oligosaccharide kit, an IEF protein mix 3.6–9.3, bovine serum albumin, crystal violet, (CV), and Coomassie brilliant blue R250 and G250 were purchased from Sigma-Aldrich (St. Louis, MO, USA). All other reagents were purchased from Sinopharm Chemical Reagent Corporation (Shanghai, China) and were of the highest analytical grade.

### 4.2. Crude Dextranase Production

Extracellular dextranase production was performed in medium containing 5 g/L yeast extract, 5 g/L peptone, 10 g/L dextran T20, and 5 g/L NaCl. The pH was adjusted to about 8.0 before autoclaving. Then, the production medium was inoculated with *Catenovulum* sp. DP03. After fermenting at 30 °C for 28 h, a cell-free culture broth was obtained by centrifugation for 20 min at 12,000× *g* and 4 °C.

### 4.3. Purification of Dextranase

First, excess water and other matter were removed from the crude dextranase product by ultrafiltration (Watson Marlow, Cornwall, UK) using a 30,000 NMWC hollow fiber cartridge (GE Healthcare) at room temperature. Deionized water was added three times. The crude enzyme was finally concentrated to one tenth of the original volume of the culture broth. Second, pre-cooled ethanol (−40 °C) was added to the crude enzyme solution slowly and agitated gently for 10 min. An ethanol: enzyme ratio of 0.6:1.2 (*v*:*v*) was found to be optimal. After centrifugation for 15 min at 12,000× *g* and 4 °C, the precipitate was dissolved in 10 mM Tris-HCl buffer (pH 7.5). Third, ammonium sulfate precipitation was performed using a magnetic stirrer. The supernatant from ethanol precipitation was placed in a beaker within an ice tray. Then 25% saturated ammonium sulfate was added to the solution, which then was allowed to stand at 4 °C for 1 h, after which it was centrifuged at 12,000× *g* at 4 °C for 20 min. The supernatant was collected, and 60% saturated ammonium sulfate was added. The mixture was allowed to stand for 1 h, and precipitated protein was collected by centrifugation 20 min at 12,000× *g* and 4 °C. Then the pellet was dissolved and dialyzed using 10 mM Tris-HCl buffer (pH 7.5). Finally, the enzyme sample was loaded onto a Q-Sepharose column (1.6 cm × 10 cm; GE Healthcare). Chromatographic analysis was conducted by a fast protein liquid chromatography (FPLC; Bio-Rad, Hercules, CA, USA) at room temperature. Proteins were eluted with several NaCl gradients in 10 mM Tris-HCl (pH 7.5) at a flow rate of 0.8 mL/min. Protein content and enzyme activity for each fraction were monitored. The enzyme-containing fractions were concentrated using ultracel-10k centrifugal filters (Millipore, Burlington, MA, USA).

### 4.4. SDS-PAGE and Isoelectric Focusing

SDS-PAGE analysis of dextranase was performed according to Laemmli [48] with minor modifications. After electrophoresis, the gel was stained with Coomassie brilliant blue R-250. Isoelectric focusing (IEF) was performed using a Pharmacia PhastGel System (GE Healthcare) on PhastGel IEF 3–9 according to the manufacturer’s instructions. An IEF protein marker (mix 3.6–9.3) was used as the pI standard.

### 4.5. Enzyme Assay and Protein Measurement

Enzyme activity was measured using dextran T70 as the substrate (3%, *m*/*v*) in 0.1 M Tris-HCl buffer (pH 8.0) using the 3,5-dinitrosalicylic acid method. Maltose served as the standard. One unit of dextranase activity was defined as the amount of enzyme capable of hydrolyzing dextran to 1 μM of reducing sugar in1 min [49]. Protein concentration was measured by the Bradford protein assay [50] using bovine serum albumin as the standard.

### 4.6. Enzyme Properties

#### 4.6.1. Effects of pH on Activity and Stability of Cadex

Dextranase activity was measured in buffers of different pH values and containing 3% dextran T70. To determine the effects of pH on dextranase stability, the enzyme solution was incubated at 25 °C for 1 h, and residual activity was measured at the optimum pH. Solutions (50 mM) with different pH values were used as follows: citrate buffer (pH 4.0–6.0), sodium phosphate buffer (pH 6.0–7.5), Tris-HCl (pH 7.5–9.0), and NaHCO_3_-Na_2_CO_3_ (pH 9.0–11.0).

#### 4.6.2. Effects of Temperature on Activity and Stability of Cadex

The temperature for the enzymatic reaction was optimized by experimentation at different temperatures (0–70 °C). To assess thermal stability, the enzyme solution was pre-heated at temperatures of 30 °C, 40 °C, and 50 °C for 0–5 h. Residual enzyme activity was measured at each interval.

#### 4.6.3. Effects of Metal Ions and Chemicals on Cadex Activity

The effects of different solutions containing chloride metal ion salts (final concentrations of 1 mM and 5 mM) and chemicals on purified Cadex activities were determined. The relative enzyme activity in the presence of metal ions and chemicals was calculated based on activity in the absence of reagent.

#### 4.6.4. Substrate Specificity

To determine the substrate specificity of Cadex, dextranase activity in the enzymatic hydrolysis of carbohydrates with various glycosidic linkages was determined using the method described by Wu.et al. [22]. Dextranase was incubated in 50 mM Tris-HCl (pH 8.0) with various carbohydrates at 40 °C for 20 min. Relative activity was expressed in percentage values of the highest activity, which was set as 100%.

#### 4.6.5. Products of Cadex Hydrolysis

First, Cadex hydrolysis took place in dextran T70 solution, and the products were analyzed by TLC using a silica gel GF254 plate developed in a chloroform: acetic: acid: water ratio of = 5:7:1 (*v*:*v*:*v*). An oligosaccharide kit was used as the standard.

Second, using the optimum dextranase temperature and pH, 3% dextran T70 samples were digested for different periods (15 min, 30 min, 1 h, 3 h, and 5 h). The products were identified and analyzed with the Waters 600 and Waters Sugar-Pak1 (300 mm × 7.8 mm; Waters, Milford, MA, USA) HPLC with a differential refraction detector. The mobile phase was water at 0.4 mL/min, the column temperature was 85 °C and the injection volume was 20 μL. The standard sugars were glucose, maltose, maltotriose, isomaltotriose, isomaltotetraose, isomaltopentose, and isomaltohexose. For quantification, the peak areas were determined. Data acquisition and processing were conducted using Empower GPC software (Waters, Milford, MA, USA).

### 4.7. Effects of Cadex on Biofilm

#### 4.7.1. Biofilm Mass Assay

The biofilm mass was assayed by CV staining according to the protocol of Cardoso et al. [51] with some modifications. Briefly, biofilms were grown on a flat bottom sterile 96-well plate (Greiner, Frickhausen, Germany) in which the cultured medium was removed. To each well, 0.2 mL of 0.2 M phosphate buffer was added three times to clean the unattached biofilms, which were left to air dry and fixed for 60 min. Then 0.2 mL of 1% CV was added to each well for 5 min. Following the staining step, the CV solution was removed and the biofilms were cleaned and dried, after which 0.2 mL of 95% ethyl alcohol was added to re-solubilize the dyed biofilms. The CV solutions were obtained and transferred to a new 96-well plate and the optical density of the content was measured using a microtiter plate spectrophotometer (Bio-Rad) at 595 nm.

#### 4.7.2. Effects of Cadex on Biofilm Formation

Base on the biofilm mass assay, MBIC of *Streptococcus mutans* ATCC 25175 (American Type Culture Collection (ATCC), Manassas, VA, USA) was measured. The effects of Cadex and a homogenetic purity dextranase from *Penicillium* (SA D8144; Sigma) on *S. mutans* biofilm formation were investigated using SEM at MBIC_90_. *S. mutans* was pre-inoculated in BHI medium without sucrose at 37 °C for 15 h. Then, 1 mL of this precultured solution was inoculated into fresh BHI medium with 1% sucrose (20 mL in 100 mL Erlenmeyer-type flask). Sterile glass coverslips were placed in the BHI medium. The media were co-cultured with *S. mutans* and incubated with Cadex at 37 °C for 3 h, 9 h, and 18 h. An identical assay with an equal volume of cell-free deionized water served as the blank control. All coverslips were collected for fixation, and were dehydrated and dried according to the procedure described by Tao et al. [52]. The coverslips were sputter-coated with gold (JFC-1600, JEOL, Tokyo, Japan) and viewed by SEM (JSM-6390LA; JEOL).

## 5. Conclusions

Cadex from the marine bacterium *Catenovulum* sp. was purified to 29.6-fold homogeneity. It showed a specific activity of 2309 U/mg protein and a molecular weight of 75 kDa. Its optimum pH and temperature were 8.0 and 40 °C, respectively. The enzyme was stable at temperatures below 30 °C and pH values within 5–11. Some mental ions and chemicals might activate Cadex, but others might inhibit it or leave it unaffected. Cadex was identified as a typical endo-dextranase. The main hydrolysis products were isomaltoogligosaccharides which may be included as a prebiotic supplement to promote the growth and proliferation of intestinal flora. Cadex inhibited biofilm formation by *S. mutans*. Thus, this alkaline- and cold-adapted dextranase from the marine bacterium *Catenovulum* sp. appears to be efficacious under both mesophilic and alkalophilic conditions, thus is a potential candidate for development into a novel marine oral biofilm removal drug.

## Figures and Tables

**Table 1:** Purification of Cadex.

Purification Step	Total Protein (mg)	Total Activity (U)	Specific Activity (U/mg)	Purification (-Fold)	Yield (%)
Culture broth	80.6	6314.3	77.9	1	100
30 kDa ultrafiltration	36.5	5973	163.5	2.1	94.6
Alcohol precipitation	20.9	4664.6	223.3	2.9	73.9
Ammonium sulfate precipitation	6.74	2303.9	341.6	4.4	36.5
Ion exchange chromatography	0.46	1069.5	2309	29.6	16.9

**Table 2:** Effects of metal ions on Cadex activity.

Reagents	Relative Activity (%) (1 mM)	Relative Activity (%) (5 mM)
Control	100 ± 1.45	100 ± 0.71
Ba^2+^	99.82 ± 0.51	97.73 ± 0.95
NH_4_^+^	99.91 ± 1.23	77.8 ± 1.8
Ca^2+^	97.46 ± 1.1	76.58 ± 2.96
Mg^2+^	102.77 ± 2.27	104.47 ± 2.91
K^+^	100.62 ± 3.69	91.87 ± 3.47
Cu^2+^	1.97 ± 0.85	2.00 ± 1.11
Fe^3+^	21.96 ± 1.67	0
Zn^2+^	50.10 ± 1.92	2.47
Li^+^	99.98 ± 0.4	90.53 ± 0.67
Cd^2+^	41.14 ± 1.29	14.55 ± 1.65
Ni^2+^	51.39 ± 1.35	21.35 ± 0.7
Co^2+^	60.99 ± 2.32	29.00 ± 1.15
Sr^2+^	103.75 ± 1.77	106.60 ± 1.89

**Table 3:** Effects of chemical treatment of dental caries on Cadex activity.

Reagents (*w*/*v*)	Relative Activity (%)
Control	100 ± 1.15
0.5% sodium lauryl sulfate	7.31 ± 1.39
0.1% sodium fluoride	93.8 ± 1.40
0.1% carboxybenzene	102.2 ± 0.32
0.1% xylitol	100.3 ± 0.50
5% ethanol	105.5 ± 0.70

**Table 4:** Substrate specificity of Cadex.

Substrate	Main Linkages	Relative Activity (%)
Dextran T20	α-1,6	90.42 ± 0.25
Dextran T40	α-1,6	91.29 ± 0.67
Dextran T70	α-1,6	95.65 ± 1.55
Dextran T500	α-1,6	100 ± 1
Soluble starch	α-1,4, α-1,6	4.93 ± 1.36
Microcrystalline cellulose	β-1,4	0
Chitin	β-1,4	0
Pullulan	α-1,4	0

**Table 5:** Content of sugar (%) in hydrolysates after enzymatic hydrolysis of dextran by Cadex.

Time of Hydrolysis	Hydrolysis Productions						
	G1	G2	G3	G4	G5	G6	G7
15 min	2.02	18.59	12.31	19.22	21.02	12.49	14.36
30 min	1.95	17.75	12.03	19.13	21.01	13.1	15.03
1 h	1.9	17.44	11.69	18.86	20.77	13.71	15.63
3 h	1.94	16.74	11.25	18.57	21.05	14.53	15.92
5 h	1.96	16.24	9.62	17.7	19.9	16.29	18.3

**Table 6:** Biofilm inhibitory rates with different concentrations of dextranase.

Concentration of *Penicillium* Dextranase (U/mL)	Biofilm Inhibitory Rate ^a^ (%)	Concentration of Cadex (U/mL)	Biofilm Inhibitory Rate ^a^ (%)
0	0	0	0
5	27.46 ± 1.28	5	33.31 ± 0.99
10	39.44 ± 1.33	10	52.39 ± 1.21
15	50.2 ± 1.42	15	62.2 ± 0.92
20	63.13 ± 0.89	20	71.3 ± 0.69
25	73.42 ± 1.13	25	85.45 ± 0.70
30	82.16 ± 0.92	30	91.11 ± 0.83
40	89.34 ± 0.93	35	94.21 ± 1.13

^a^ The biofilm inhibitory rate was calculated at an absorbance of 595 (A_595_) of the crystal violet stained biofilm without dextranase subtracted from A_595_ of biofilm with dextranase, and divided by A_595_ of biofilm without dextranase multiplied by 100%.
